# Primary Pancreatic Leiomyosarcoma: A Rare Malignant Transformation of Primary Pancreatic Head Leiomyoma

**DOI:** 10.7759/cureus.19328

**Published:** 2021-11-07

**Authors:** Kislay Kant, Pradeep Rebala, Guduru V Rao, D N Reddy

**Affiliations:** 1 Surgical Gastroenterology, Asian Institute of Gastroenterology, Hyderabad, IND; 2 Gastroenterology, Asian Institute of Gastroenterology, Hyderabad, IND

**Keywords:** pancreatic malignancy, pancreatic neoplasm, primary pancreatic mesenchymal tumor, primary pancreatic leiomyosarcoma, primary pancreatic leiomyoma

## Abstract

Primary pancreatic leiomyosarcoma and primary pancreatic leiomyoma are rare tumors of the pancreas. Primary pancreatic leiomyoma is a benign tumor and its conversion to leiomyosarcoma has never been reported. We report a case of malignant transformation of pancreatic leiomyoma. In this case, a 75-year-old male, who presented with a mass in the head of the pancreas, was diagnosed with primary pancreatic leiomyoma. The patient maintained well on symptomatic treatment for 13 years. However, later the patient presented with loss of appetite, significant weight loss, and an abdominal lump, which was diagnosed to be locally advanced primary pancreatic leiomyosarcoma. The patient was provided the best supportive care and died after 11 months of diagnosis. Hence, we conclude that a more radical treatment approach is needed in patients with primary pancreatic leiomyoma.

## Introduction

Primary mesenchymal tumors of the pancreas are rare, with only 221 cases reported to date [1]. Primary pancreatic leiomyosarcomas are an even rarer subtype, with only 69 reported cases to date [2]. Herein, we report the first case of a patient with pancreatic leiomyoma which underwent a malignant transformation to leiomyosarcoma.

## Case presentation

A 75-year-old man presented to our institution with a 40-year history of dyspeptic symptoms. Physical examination was unremarkable. Contrast-enhanced computed tomography (CECT) of the chest, abdomen, and pelvis revealed a 4 × 3-cm well-circumscribed mass localized in the head of the pancreas. This tumor showed peripheral enhancement in the early arterial phase and homogenous enhancement in the late arterial phase. The pancreatic duct was not dilated, surrounding vessels were free, and there was no evidence of any nodal or distant metastasis. Core needle biopsy of the mass revealed the presence of spindle cells without any atypia. Two years before presenting to our institution, the patient presented to another center for the same complaint, where he underwent CECT of the abdomen, which revealed a 4 × 3-cm mass in the head of the pancreas. Based on the presence of a pancreatic head mass of constant size for two years and core needle biopsy showing spindle cells, the patient was diagnosed with primary pancreatic leiomyoma and was advised to be under follow-up. He was given symptomatic treatment for dyspepsia, to which he responded well. During yearly follow-up examinations for the next eight years, which included imaging studies, the mass size did not change and the patient remained asymptomatic. The patient was then lost to follow-up.

**Figure 1:**
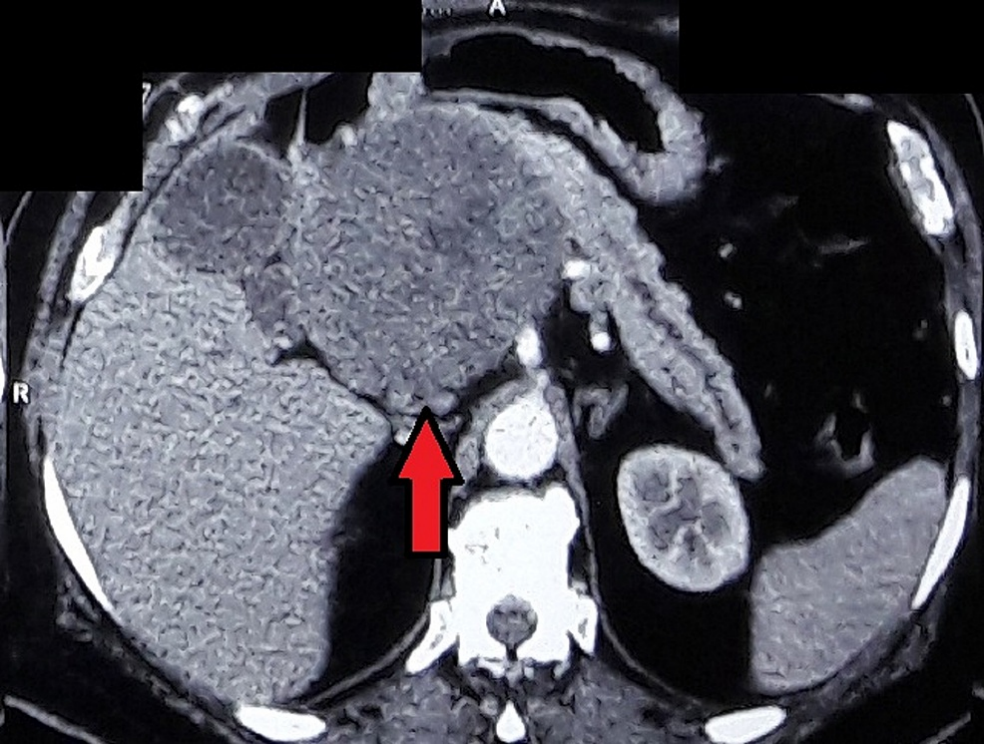
Contrast-enhanced computed tomography of the abdomen showing a 15 × 13 × 10-cm mass (red arrow) in the pancreatic head accompanied with a dilated pancreatic duct.

Thirteen years after the initial diagnosis of the pancreatic mass, the patient presented with a three-month history of loss of appetite, significant weight loss, and a lump in the right upper abdomen. Physical examination revealed a 10 × 12-cm hard, nodular mass in the right hypochondrium, which did not move with respiration. Hemogram and liver function tests were normal. Abdominal ultrasound examination three months before the latest admission revealed a 13 × 10-cm solid mass in the pancreatic head. Abdominal CECT at admission revealed a 15 × 13 × 10-cm mass with heterogenous enhancement in late phase involving the pancreatic head with main portal vein encasement and dilated pancreatic duct and common bile duct (Figure 1).

**Figure 2:**
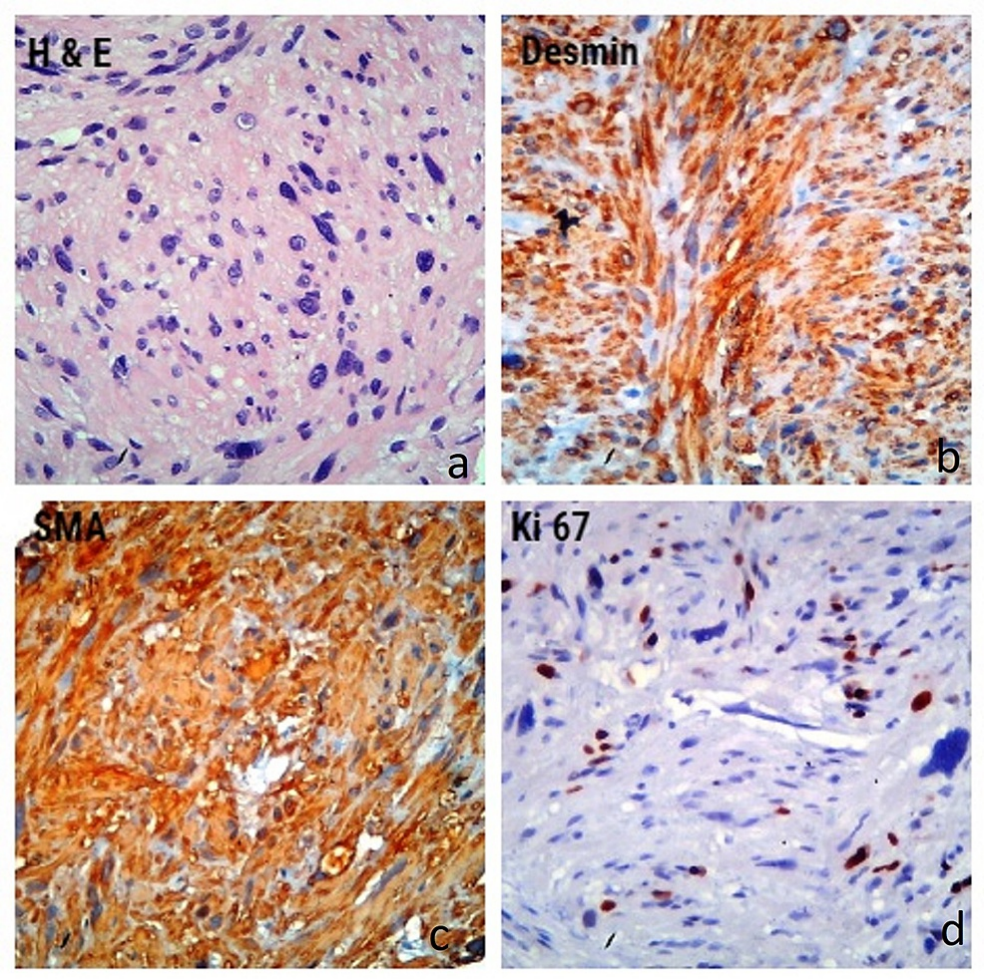
(a) Histopathological examination of the biopsy specimen showing spindle cells (H&E). Immunohistochemical staining shows that the biopsy specimen is positive for (b) desmin and (c) smooth muscle actin. (d) The Ki-67 index is 20%. H&E: hematoxylin/eosin staining; SMA: smooth muscle actin

Immunohistochemical evaluation revealed that the biopsy samples collected by ultrasound-guided fine-needle aspiration were positive for smooth muscle actin and desmin but negative for c-Kit, DOG-1, CD34, and S-100. The Ki-67 index was 20%. These results led to the diagnosis of primary pancreatic leiomyosarcoma (Figure 2). The patient could not tolerate chemotherapy including doxorubicin and ifosfamide and received best supportive care. The patient died 11 months after the leiomyosarcoma diagnosis.

## Discussion

The guidelines for the management of pancreatic mesenchymal tumors, which are rare neoplasms, are based on the few case reports reported to date. Pancreatic mesenchymal tumors include fibromatoses, cavernous hemangioma, schwannoma, solid and cystic hamartoma, solitary fibrous tumor, inflammatory myofibroblastic tumor, angiomyolipoma, and sarcoma (undifferentiated/unclassified sarcoma, leiomyosarcoma, atypical lipomatous tumor/well-differentiated liposarcoma, Ewing sarcoma/primitive neuroectodermal tumor) [3]. Amongst all pancreatic neoplasm, leiomyosarcoma accounts for only 0.1% [4].

Presenting symptoms of primary pancreatic leiomyosarcomas may include abdominal pain, abdominal mass, weight loss, and jaundice, although the diagnosis is usually incidental [1,5,6]. Pancreatic leiomyosarcomas may be located in the pancreatic head, body, or tail [1,5]. These tumors are usually large in size at presentation [6]. CECT characteristics of leiomyosarcomas include a heterogeneous mass with or without necrosis or calcification [2]. In a previous study using MRI, it was shown that pancreatic leiomyosarcomas appear isointense with skeletal muscle on T1-weighted images, hyperintense on T2-weighted images, and exhibit heterogeneous enhancement following intravenous gadolinium like most leiomyosarcomas [2]. By light microscopic evaluation, pancreatic leiomyosarcomas comprise interlacing bundles and fascicles of spindle cells [5]. Immunohistochemically, pancreatic leiomyosarcomas are usually positive for smooth muscle actin and desmin and negative for c-kit, S-100, and cytokeratin [5].

About 25% of pancreatic leiomyosarcomas may already have distant metastases at the time of diagnosis, and 19% of patients present with local infiltration, similar to that observed in the present case [6]. Lymph node metastasis is an extremely rare finding in patients with pancreatic leiomyosarcoma [2,5,6].

For resectable pancreatic leiomyosarcomas, radical resection is the treatment of choice and may include pancreaticoduodenectomy or distal pancreatectomy. The median survival duration from the time of diagnosis is 48 months [6]. Distant metastasis, local invasion, and non-radical resection are markers of poor outcomes in patients with pancreatic leiomyosarcomas [6].

Our extensive literature search did not reveal other reported cases of patients with leiomyoma that underwent a malignant transformation to leiomyosarcoma. In the present case, leiomyoma was diagnosed on the basis of imaging and core needle biopsy performed at the initial presentation. The well-documented aggressive nature of leiomyosarcoma and the complete absence of symptoms during the follow-up period do not lend strong support for the possibility that the mass at initial presentation 13 years ago was leiomyosarcoma. This unique case of pancreatic leiomyoma that underwent malignant transformation suggests that a more radical treatment approach might be considered for pancreatic leiomyomas with no malignant potential.

## Conclusions

The guidelines for the management of pancreatic leiomyoma and leiomyosarcoma, rare neoplasms of the pancreas, are based on the few reported cases. Malignant transformation should be considered in patients with pancreatic leiomyoma. Pancreatic leiomyosarcoma is prone to distant metastasis, and local invasion and radical resection is the only curative option available in resectable cases. Thus, surgery should be considered in patients with pancreatic leiomyomas with no malignant potential.
